# Draft Genome Sequence of *Bordetella bronchiseptica* KU1201, the First Isolation Source of Arylmalonate Decarboxylase

**DOI:** 10.1128/genomeA.00373-15

**Published:** 2015-05-07

**Authors:** Shosuke Yoshida, Junichi Enoki, Risa Hemmi, Robert Kourist, Norifumi Kawakami, Kenji Miyamoto

**Affiliations:** a Department of Biosciences and Informatics, Keio University, Yokohama, Japan; b Faculty for Biology and Biotechnology, Ruhr-University, Bochum, Germany

## Abstract

The analysis of the 6.8-Mbp draft genome sequence of the phenylmalonate-assimilating bacterium *Bordetella bronchiseptica* KU1201 identified 6,358 protein-coding sequences. This will give us an insight into the catabolic variability of this strain for aromatic compounds, along with the roles of arylmalonate decarboxylases in nature.

## GENOME ANNOUNCEMENT

*Bordetella bronchiseptica* KU1201 (formerly, *Alcaligenes bronchisepticus* KU1201) was isolated from soil sample through enrichment culture on phenylmalonate as a sole carbon source (1). We determined that this strain harbors a novel unique enzyme that shows decarboxylation activities to arylmalonates, materials unfound in the natural environment. The enzyme AMDase catalyzes an asymmetric decarboxylation of prochiral arylmalonates to produce optically pure (R)-arylcarboxylates without any cofactor (2). Thus far, homologous AMDases from different sources have been characterized (3, 4). However, physiologically true substrate(s) catalyzed by AMDase and the metabolic pathway that this enzyme mediates are still uncovered. Genomic DNA of *Bordetella bronchiseptica* KU1201 was obtained using a Wizard genomic DNA purification kit (Promega, Madison, WI), followed by further purification by phenol:chloroform:isoamyl alcohol (25:24:1, vol/vol) extraction. Shearing and library preparation were done in accordance with Illumina’s TruSeq DNA PCR-Free Sample Preparation Guide (5). The whole-genome shotgun sequence of *Bordetella bronchiseptica* KU1201 was obtained by an Illumina GAIIx run.

Sequencing yielded 22,785,092 single reads with an average length of 119 bp. Reads were trimmed and quality filtered using CLC Genomics Workbench version 6.5.1. After processing, 22,741,361 reads remained. Assembly of the reads was carried out on CLC Genomics Workbench version 6.5.1. The G+C content of the assembled contigs is 66.1%. The draft genome comprises 90 contigs, with an *N*_50_ value of 248,258 bp. The total length is 6.8 Mbp. Annotation was carried out by uploading the generated contigs to the RAST server (6), which found 6,358 protein-coding sequences (CDSs) comprising 2,969 genes in subsystems. A total number of 55 RNA genes were detected by RAST analysis. Two hundred twelve genes are involved in the metabolism of aromatic compounds, including 144 genes for the central ring-cleavage pathway and 37 genes for the peripheral channeling pathways, suggesting the versatility in aromatic catabolism of this strain.

### Nucleotide sequence accession numbers.

This whole-genome shotgun project has been deposited in DDBJ/EMBL/GenBank under the accession numbers BBVB01000001 to BBVB01000090 (BioProject number PRJDB3555).
